# A Hybrid Stacked CNN and Residual Feedback GMDH-LSTM Deep Learning Model for Stroke Prediction Applied on Mobile AI Smart Hospital Platform

**DOI:** 10.3390/s23073500

**Published:** 2023-03-27

**Authors:** Bassant M. Elbagoury, Luige Vladareanu, Victor Vlădăreanu, Abdel Badeeh Salem, Ana-Maria Travediu, Mohamed Ismail Roushdy

**Affiliations:** 1 Faculty of Computer and Information Sciences, Ain Shams University, Cairo 11566, Egypt; 2 Institute of Solid Mechanics of the Romanian Academy, 010141 Bucharest, Romania; 3 Faculty of Computers and Information Technology, Future University in Egypt, New Cairo 11835, Egypt

**Keywords:** artificial intelligence, mobile health, stroke monitoring, iomt-stacked convolutional neural networks, GMDH neural networks, Deep LSTM, biomedical EMG signal processing

## Abstract

Artificial intelligence (AI) techniques for intelligent mobile computing in healthcare has opened up new opportunities in healthcare systems. Combining AI techniques with the existing Internet of Medical Things (IoMT) will enhance the quality of care that patients receive at home remotely and the successful establishment of smart living environments. Building a real AI for mobile AI in an integrated smart hospital environment is a challenging problem due to the complexities of receiving IoT medical sensors data, data analysis, and deep learning algorithm complexity programming for mobile AI engine implementation AI-based cloud computing complexities, especially when we tackle real-time environments of AI technologies. In this paper, we propose a new mobile AI smart hospital platform architecture for stroke prediction and emergencies. In addition, this research is focused on developing and testing different modules of integrated AI software based on XAI architecture, this is for the mobile health app as an independent expert system or as connected with a simulated environment of an AI-cloud-based solution. The novelty is in the integrated architecture and results obtained in our previous works and this extended research on hybrid GMDH and LSTM deep learning models for the proposed artificial intelligence and IoMT engine for mobile health edge computing technology. Its main goal is to predict heart–stroke disease. Current research is still missing a mobile AI system for heart/brain stroke prediction during patient emergency cases. This research work implements AI algorithms for stroke prediction and diagnosis. The hybrid AI in connected health is based on a stacked CNN and group handling method (GMDH) predictive analytics model, enhanced with an LSTM deep learning module for biomedical signals prediction. The techniques developed depend on the dataset of electromyography (EMG) signals, which provides a significant source of information for the identification of normal and abnormal motions in a stroke scenario. The resulting artificial intelligence mHealth app is an innovation beyond the state of the art and the proposed techniques achieve high accuracy as stacked CNN reaches almost 98% for stroke diagnosis. The GMDH neural network proves to be a good technique for monitoring the EMG signal of the same patient case with an average accuracy of 98.60% to an average of 96.68% of the signal prediction. Moreover, extending the GMDH model and a hybrid LSTM with dense layers deep learning model has improved significantly the prediction results that reach an average of 99%.

## 1. Introduction

The proposed architecture aims to develop, analyze and incorporate artificial intelligence and deep learning technology and extend our previous research on mobile AI telemedicine platforms [1] to harness the findings of research and development in the fields of biomedical signal processing (ECG, EMG/ECG). In the sense of emergency, artificial intelligence, and tracking in a healthcare setting, this article is intended to create adaptive, collaborative, and creative artificial intelligence and intelligence technologies for patients.

Artificial intelligence (AI) technologies in Smart health living hospitals for connected and mobile health AI edge computing integrated with telemedicine systems have opened up new opportunities in healthcare systems and complex diseases. Predictive analytics [1,2,3,4] and intelligent mobile edge computing in healthcare [5,6,7,8,9] help patients manage their treatments, especially for stroke monitoring and predictive analytics [5,9], which is a complex problem due to real-time detection of patient cases and real-time biomedical sensor signal streaming of each person independently. The intelligent mobile health application aims to help stroke patients record their episode once it occurs based on EMG signals. However, classifying real-time EMG signals [10,11,12] is a complex task, especially due to problems with patient muscle signal feedback. This research paper introduces a new predictive analytics model for stroke prediction using technologies of mobile health, and artificial intelligence algorithms such as stacked CNN, GMDH, and LSTM models [13,14,15,16,17,18,19,20,21,22]. A new prototype of a mobile AI health system has also been developed with high-accuracy results, which are going to be discussed in this paper. The main motivation is automating classification and intelligent emergency assistance for patients who suffer strokes.

Deep learning (multiple layer neural networks) enables end-to-end learning, where higher dimensional features (e.g., the correlation between multi-bio signal measurement datasets) are input directly to the neural network. IoMT devices such as ECG, and EMG send information directly so that signals can be analyzed and used as input for mobile devices and intelligent telemedicine platforms.

In addition, stroke prediction research is still missing a real-time AI-based heart diagnosis and stroke prediction system to be developed as an AI-based platform to be used, especially in the new era of smart hospitals and artificial intelligent technologies in European hospitals [23,24,25,26,27,28,29,30,31].

The experiments presented in this paper discuss the measurements of the EMG dataset and signal prediction results. The focus is on using IoMT implemented within the framework of a novel deep learning telemedicine platform for an AI smart hospital setting that can deliver care to stroke patients. This platform can be used as a portable patient/person assistive emergency tool and as a telemedicine hospital support system as well as an inter-hospital support system for larger hospital associations due to the flexible system model. Several deep learning models have been introduced in research [32,33,34,35,36,37,38,39,40], targeting cardiovascular and stroke diseases.

The experiments presented in this paper discuss the measurements of the EMG dataset and signal prediction results. We are focused to use IoMT implemented within a framework of novel deep learning telemedicine platform for AI smart hospital settings that can deliver care to stroke patients and people in a smart health environment. This work proposed the following artificial intelligence platform and deep learning techniques applied for stroke patients’ emergencies:

1. An innovative automated proposed biomedical deep learning cloud platform for stroke patients’ emergencies and remote using stacked convolutional neural networks the proposed solution offers complete intelligent healthcare services inside homes, for elderlies, families, and emergency care services. The main goal is heart stroke prediction, monitoring, and diagnosis. The AI-connected health platform includes deep learning models to the cloud and a mHealth module to send alerts.
2. The innovative artificial intelligence telemedicine platform for stroke prediction and emergency situations. That depends on statistical methods for EMG signal tracking and prediction such as group handling methods (GMDH) neural network [8,9] for patient stroke real-time prediction. The GMDH deep learning model is further enhanced with LSTM deep learning module [18,19].
3. A new real-time CNN-stroke and heart and BAN-IOT: a deep learning model for signal deep feature extraction and classification within big data streaming environment
4. A new mobile AI engine prototype has been developed and tested for the proposed AI techniques.

The paper consists of four sections as follows: the introduction consists of background and related work. Section 2 presents related works, Section 3 discusses materials and methods used, explains EMG signal processing features extraction, explains the stacked CNN deep learning technique, and presents the usage of GMDH neural networks for stroke prediction, along with extended LSTM prediction results. Section 4 presents experimental results and the simulated AI mobile app and Section 5 discusses conclusions and future work.

## 2. Related Works

AI has been in development for decades, but only recently become good enough for people to notice, mostly due to advances in other industries besides health care. The rise of intelligent machines is approaching, and the world, especially the healthcare industry, is far from prepared for what is to come. Mobile health [19,24] applications are receiving increased attention largely due to the global penetration of mobile technologies. It is estimated that over 85% of the world’s population is now covered by a commercial wireless signal, with over 5 billion mobile phone subscriptions [11]. Tarik Taleb et al. [12] present a study on MEC (mobile edge computing) [9,19,24,33] that discusses the major enabling technologies in this domain. It explores MEC deployment considering both the perspectives of individual services as well as a network of MEC platforms supporting mobility. It also delves into an analysis of a MEC. reference architecture and its main deployment scenarios that can offer multitenancy support for application developers. R. Yongbo Li et al. [14] have developed MobiQoR: for Pushing the Envelope of Mobile [9,19,20,24,33] Edge Computing Via Quality-of-Result Optimization. Fang, S.H. et al. [16] proposes a deep learning mechanism to identify the transportation modes of smartphone users. The proposed mechanism is evaluated on a database that contains more than one thousand hours of accelerometer, magnetometer, and gyroscope measurements from five transportation modes including still, walking, running, bike, and vehicle.

Oguz Karan [5], presented an ANN model applied to smartphones to diagnose diabetes. In this study, a three-layered multilayer perceptron (MLP) feedforward neural network architecture was used and trained with the error backpropagation algorithm. Peter Pes [6], developed a smartphone-based decision support system (DSS) for the management of type 1 diabetes in order to improve quality of life. Jieun Kim [16], proposed a case-based reasoning [19,24,41,42,43,44,45,46,47,48] approach to matching the user needs and existing services, identifying unmet opportunistic user needs, and retrieving similar services with opportunities based on Apple smartphones. Swapna et al. [38] have worked on EEG signal generation and heart rate in cardiac diseases, however, they did not address stroke prediction issues. Complications of acute ischemic stroke from a medical perspective, but without addressing prediction issues were addressed. Park et al. [39] have developed an intelligent stroke monitoring system during sleeping cases only but not for outdoor multi-event systems. Aminova et al. [40] have developed a single-channel EEG predictor for cognitive function after stroke and not using EMG as a pre-stroke prediction system. Z. Yang [41] proposed a model of an IoT-cloud [42,45] based wearable ECG monitoring system for smart healthcare. Satija et al. [48] presented continuous cardiac health monitoring with signal quality-aware IoT-enabled [42,43,47,48] ECG telemetry system. Ihsanto et al. [7] proposed depthwise separable convolutional (DSC) NNs for the cardiac arrhythmia categorization. The MIT-BIH arrhythmia database was utilized for the assessment of the proposed ensemble CNNs’ performance. The proposed algorithm could classify the data into sixteen classes. In addition to that, the sensitivity was 99.03%, specificity was 99.94%, positive predictive was 99.03%, and accuracy was 99.88%. Predictive analytics in healthcare decision-making [3,47,48] deals with information retrieval to predict an unknown event of interest, typically a future event. Using technology that learns from data to predict these unknown events could drive better decisions.

## 3. Materials and Methods

**Figure 1:**
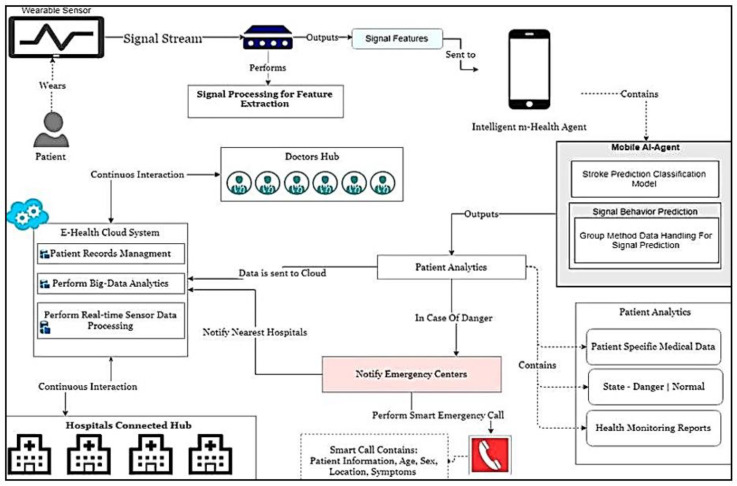
The artificial intelligence architecture for smart hospital connected health.

The architecture of the proposed intelligent hospital for the connected health modules is presented in Figure 1. It consists of a wearable sensors module that sends signal streams for signal processing modules and mobile AI health for stroke prediction. It can be connected to the cloud, as seen in the following figure, in order to accomplish effectively the aims of the research. The smart hospital stroke system, which integrates recent advances in artificial intelligence and predictive computing with telemedicine applications, is a continuously growing field in telemedicine. A stroke can cause sudden death and is a matter of urgency. It is one of America’s leading causes of death. Heart/brain stroke, for these reasons, is an emergency and must be treated promptly before any complications occur. Recent research shows that a smart hospital heart/stroke system is at the forefront of current research, especially in the field of chronic diseases and emergency conditions such as heart attacks. Today, however, an intelligent patient control screening device is lacking. In addition, such a system needs smart algorithms for patient stroke prediction and emergencies to warn better diagnostic decisions and fast patient care response in the process. In this paper, a modern intelligent hybrid architecture is proposed. The most important activities and actions in this innovative architecture for smart hospital-connected health approach are concentrated around the individual person/patient. 

The proposed Mobile AI Health Agent receives all necessary inputs from medical sensors, and sensors operating on EMG, as will be explained in this article, but it can be extended to other biomedical sensors for stroke and heart diseases [24]. On the other hand, after the sensors’ data and information are processed and results are obtained, they are delivered to the individual under tracking.

**Figure 2:**
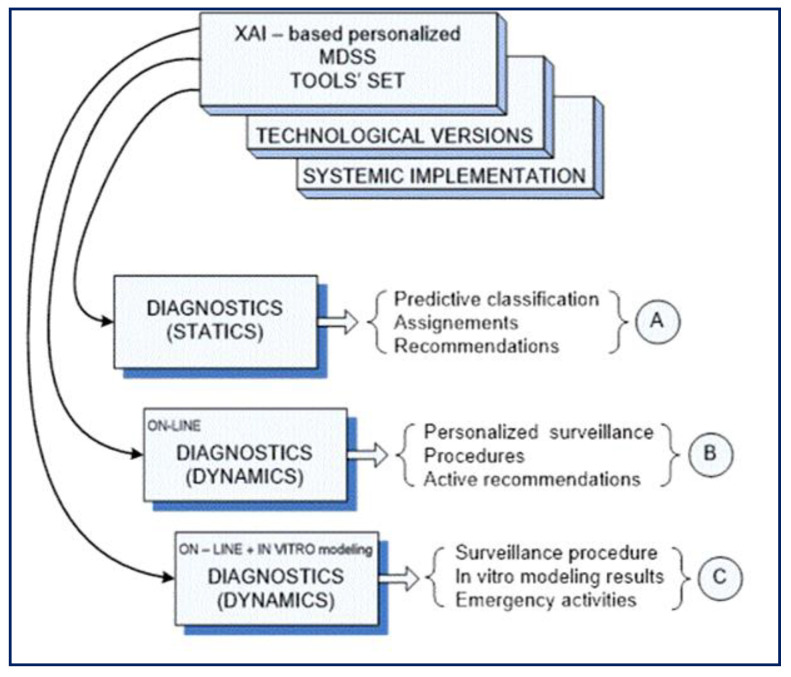
Concept of the XAI-based HC tools for MDSS. Blocks A, B and C are described in details in the related paragraphs in this section.

The proposed architecture of Figure 1 is also integrated with set of DSS tools for explainable artificial intelligence (XAI)-based human-centric (HC) applications, as shown in Figure 2, where a medical decision support system (MDSS) is proposed. It is directed to cover concrete individualized needs of the patient under treatment, medication, or social care, as well as on-hand competence of concrete sub-division in a medical institution taking care of this personal case. Here, the block of comparatively static diagnostics contains a personalized toolset dedicated to primary diagnosis, predictive state classification, assignments, and individual recommendations (A), based on a set of DSS tools built on the basis of XAI.

The next block of dynamic diagnostics online is dedicated to individualized observation of the individual under permanent healthcare service and is responsible for personalized surveillance, all prescribed procedures, and active recommendations (B) for both actors: the patient, and health professionals as well.

The third block is similar to the second, but it is equipped with additional tools for offline modeling of proposed procedures in case any uncertainty appears. So, the output of this block (C) contains all recommended procedures of surveillance, results of the in vitro modeling, and recommendations concerning all possible emergency activities.

**Figure 3:**
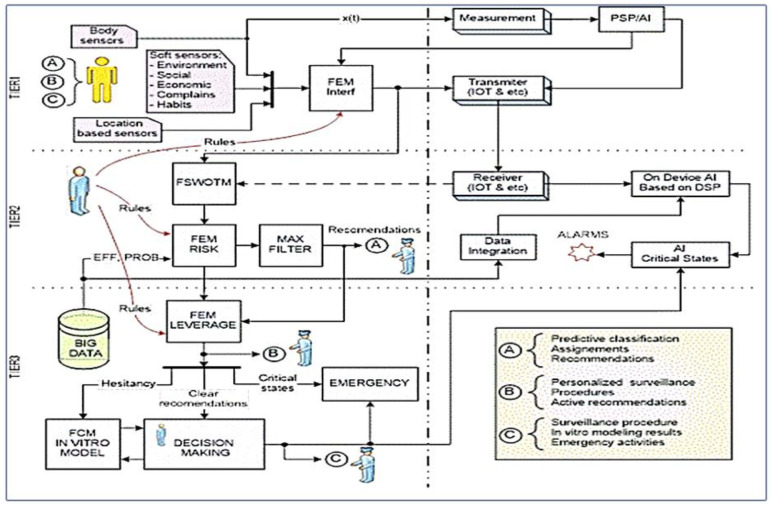
XAI general architecture consists of TIER1, TIER2, and TIER3. TIER 1 has been implemented as AI software modules for the proposed mobile AI smart hospital system.

Figure 3 illustrates the relation between the XA_ tools concept and it is integrated into our mobile AI smart hospital platform, where all digital data and evaluations are ready to be analyzed and pre-processed using deep learning [4,11,13,14,15] and feature extraction methods [28] for both Artificial intelligence telemedicine for smart ai hospital heart/stroke health units and IoT-edge cloud AI biomedical sensors processing [23,24,25,28]. On the other hand, after the sensors’ data and information are processed and results are obtained, they are delivered to the individual under tracking (A), this is where this paper presents most of the research article. The next block of XAI architecture dynamic diagnostics online is dedicated to individualized observation of the individual under permanent health care service and is responsible for personalized surveillance, all prescribed procedures, and active recommendations (B) for both actors: a patient, and health professionals as well.

The main and most important activities and actions in this human-centric approach are concentrated around the individual on TIER 1 (on the left side of Figure 3). Here, DSS receives all necessary inputs from ordinary body sensors, sensors operating on information based on individual’s location (location-based sensors) and for the first time involved Soft sensors dealing with information about environment. TIER 2 consists of fuzzy logic modules, which are not yet implemented in this paper. It will be considered for future work. 

### 3.1. Mobile AI Smart Hospital Platform: Artificial Intelligence Materials and Methods for Stroke Prediction at Home Care Emergencies Scenarios

**Figure 4:**
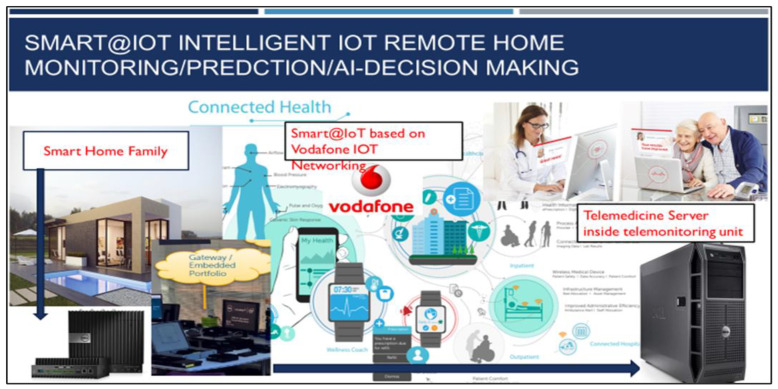
Ubiquitous intelligent IOT smart health/brain stroke deep learning platform.

An innovative automated biomedical deep learning cloud platform for stroke patients’ emergencies and remote monitoring is presented in this section. Figure 4 illustrates the possible implementation of the system for home care stroke emergencies. Personalized early risk detection and intervention solutions for prevention and treatments based on early risk detection are paramount for people facing increased health and social risks. As shown in Figure 3, the proposed solution offers complete intelligent healthcare services inside homes, for the elderly, families, and emergency care services. The main goal is for heart/brain stroke prediction, monitoring, and diagnosis. The platform [24] includes deep learning models and a mHealth module to send alerts. Two important techniques, stacked convolutional and pooling layers for biomedical sensors signal correlations are presented in this section.

### 3.2. Stacked Convolutional and Pooling Layers for Biomedical Sensors Signal Correlations

**Figure 5:**
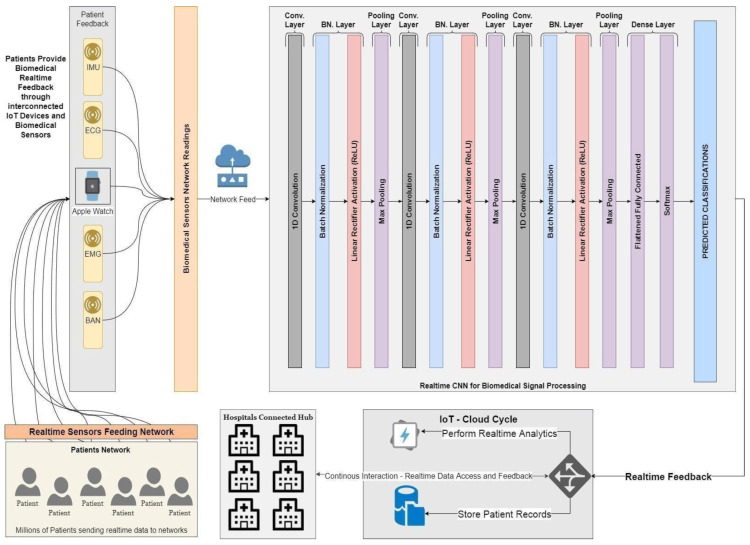
Proposed deep learning architecture: stacked convolutional and pooling layers for biomedical sensors signal correlations.

This section exploits the usage of convolutional layers, as shown in Figure 5 and their ability to extract several activation maps per signal, thus enabling us to deeply extract the correlated signal features.

Thus, the usage of stacked convolutional and pooling layers has been introduced that act as deep convolutional networks for extracting hidden signal features. A Softmax layer is then added to evaluate the model’s ability to classify input signals. The extracted features can later be used as input to another learning model that will act directly on the constructed feature vectors. Figure 5 illustrates the architecture of the tested CNN deep learning model.

The model has been tested in a scenario with a significant number of patients, sending biomedical real-time feedback through interconnected IoT devices and biomedical sensors, such as EMG, ECG, BAN, and IMU signals. The CNN model architecture is given as follows: 

Convolutional Layer: It aims to extract deep features of the input signal through several activation filters. The network extracts deep features by applying 1-D filters through the input signal and then outputs a different shape of the input signal. Pooling Layer: It aims to lower the dimensionality of the big data streamed to the convolutional layer and its output, reduces computation time, and helps the network converge. Max pooling has been used along with batch normalization. Fully Connected Layer: It flattens the output from higher dimensions down through a fully connected network of neurons, and then reduces its dimension. Multiple fully connected layers are used to feed the Softmax experimental classifier. The proposed stacked CNN network time measurements are shown in Table 1.

#### Datasets Used

Dataset Name: EMG Lower Limb Dataset

The EMG Lower Limb dataset includes different 24 patients, performing three different actions, each patient is classified as binary normal, and abnormal.

Dataset Characteristics:
Signal TypeTime SeriesNumber of Instances per Channel~12,000 SampleNumber of Channels5 Channels

Dataset Name: mHealth Dataset

The mHealth dataset includes 10 different subjects performing 12 different actions, it also includes different measurements of subject kinematic information.

Dataset Characteristics:
Signal TypeTime SeriesNumber of Instances per Channel~160,000 SampleNumber of Channels24 Channels

EMG Physical Action dataset [48]

EMG dataset contains

4 subjects

2 main binary classes normal and aggressive

10 human activities 

Aggressive: elbowing, front kicking, hammering, heading, kneeing, pulling, punching, pushing, side kicking, slapping 

Normal: bowing, clapping, handshaking, hugging, jumping, running, seating, standing, walking, waving

Almost 10,000 samples for each activity

Number of features: 8 muscles 

R-Bic, R-Tri, L-Bic, L-Tri, R-Thi, R-Ham, L-Thi, L-Ham

### 3.3. Stroke Prediction Using GMDH-Type Neural Network Enhanced with LSTM Module

This section describes the use of group method data handling (GMDH) to predict the value of the signal time series. For this, a multi-layered parametric iteration GMDH algorithm with polynomial reference functions is implemented. 

**Figure 6:**
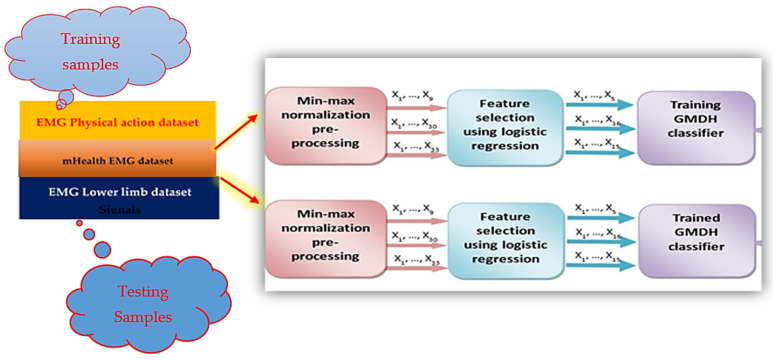
GMDH training preprocessing.

**Figure 7:**
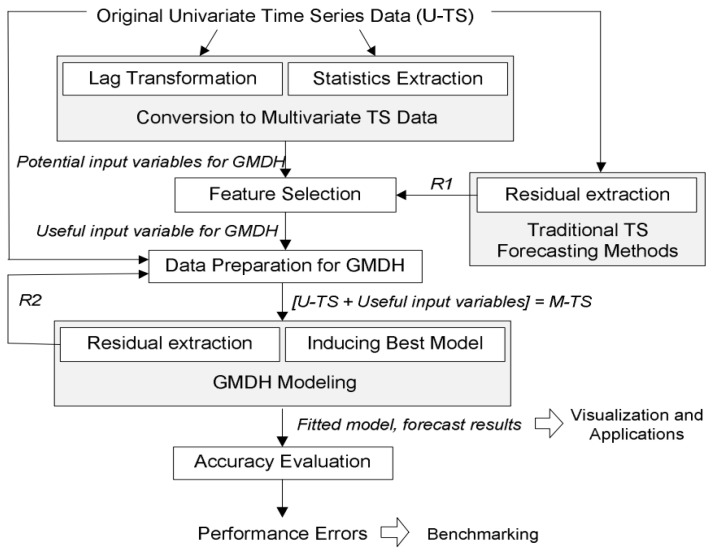
GMDH data preprocessing.

It is a sorting out of gradually complex models generated from Kolmogorov–Gabor polynomial (Figure 6 and Figure 7).

**Figure 8:**
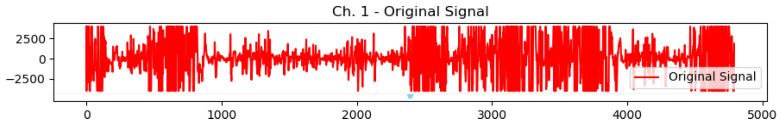
Sample of the original EMG signal.

We transform the input signal into a supervised problem, therefore making the GMDH able to predict its behavior. The graph below (Figure 8) shows a sample signal that will be later transformed into a supervised problem to feed the neural network.

The best model is chosen by the minimum of a specified external criterion characteristic, thus making it equivalent to an artificial neural network with polynomial activation functions for neurons.

The values of the previously shown signal are then transformed into a supervised problem as follows:

$$[x_{i-1},x_{i}]\to [y]\;|\;where\;y=x_{i+1}$$

We then feed the resulting feature matrices and their corresponding labels to the neural network to start the training process. The developed GMDH deep learning model [30] is built based on the reference function, then during the training process based on the selected external criterion the neurons are eliminated during the training process and the best model is created.

A selection criterion exists to perform the neurons dropout at each layer thus performing the select-and-drop training process. Selection criteria are: validation score, bias, validation score, and bias. As seen in the graph, specific neurons are selected based on the criterion and are dropped out in the next layer training process.

The training process of the neural network is stopped based on two criteria: error is not decreasing anymore, or the neural network has reached its maximum number of layers.

Least mean squared error (LMSE) is used as a loss function. The maximum number of layers for the network is defined externally before the training process.

The resulting feature matrices and their corresponding labels are then fed to the neural network to start the training process. Several transfer functions are available for the polynomial neural network; the transfer function is used as an activation function for regression problems using GMDH-type neural network. A GMDH can formulate an optimization of the structure based on the current transfer function, each transfer function is also adaptively created by another self-organizing process.

### 3.4. A Proposed Hybrid LSTM with Dense Layers Deep Learning Model for Stroke Prediction

**Figure 9:**
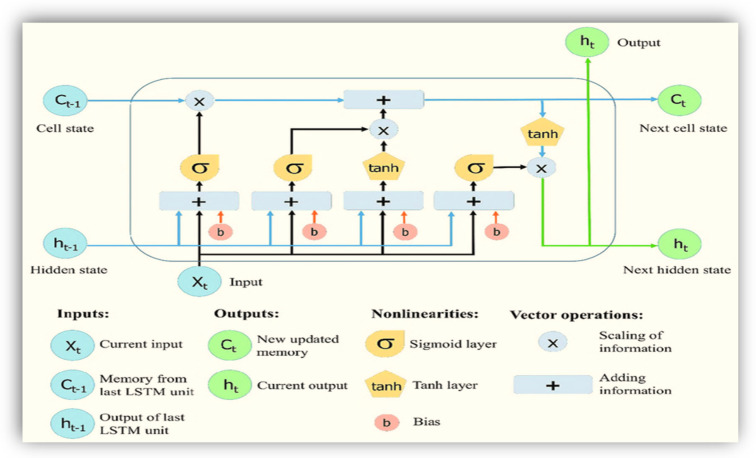
Basic LSTM deep learning gates [15,22].

LSTM is a special kind of RNN [15,22], which shows outstanding performance on a large variety of problems. It maintains state (memory) across very long sequences, basic architecture is shown in Figure 9, because LSTM is very sensitive to the data ranges we applied data normalization and scaling in the input and output. We used standard scaling for the input. It can be solved using linear activation in the output layer.

**Figure 10:**
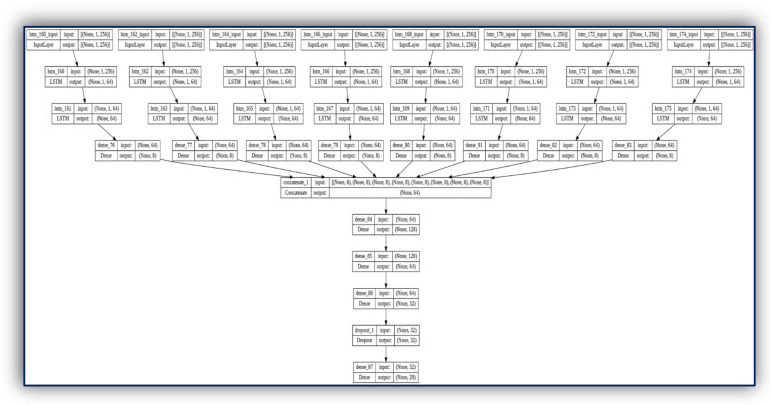
Hybrid LSTM and dense deep learning layers for EMG signals with 8 channels input.

A novel Stroke prediction algorithm is proposed based on EMG signals prediction. The proposed solution employs a novel architecture consisting of multiple LSTM recurrent dense networks as shown in Figure 10. Experimental evaluations show superior EMG prediction performance compared to previous works. Measurements with different deep learning methods such as combining CNN with LSTM show that the proposed algorithm meets performance requirements for continuous and real-time execution on IoMT devices. In contrast to many GMDH deep learning-based approaches, the proposed algorithm is lightweight for the proposed mobile AI engine, and therefore, brings continuous diagnosis and prediction with accurate GMDH–LSTM-based EMG signal prediction to IoMT simulated inputs. 

The first part chooses whether the information coming from the previous timestamp is to be remembered or is irrelevant and can be forgotten. In the second part, the cell tries to learn new information from the input to this cell. At last, in the third part, the cell passes the updated information from the current timestamp to the next timestamp.

In this article, we have built a hybrid LSTM model concatenated with dense layers. The LSTM modules are based on the basic parts of LSTM gates, each of which consists of three parts, the first part is called forget gate, the second part is known as the input gate and the last one is the output gate [15,22].

The detailed hybrid model LSTM/dense deep learning model is shown in Figure 10. For more illustrations as shown below. Its input_output takes all the EMG 8 channels of the EMG physical action dataset [47,48]. Our input features shape is (1, 256) for each 8-muscle signal. 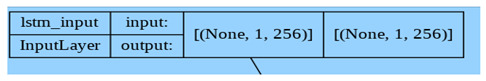


In addition, output classes are one-shot encode vectors with shape (None, 20). 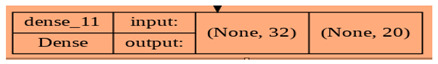


LSTM Training Results:

- Hyperparameters
  - Batch size = 256
  - Epochs = 100
  - Starting learning rate = 0.0001
- After 100 epochs from the 50
  - Training loss: 0.1890
  - Training accuracy: 0.9365
  - Validation loss: 0.2364
  - Validation accuracy: 0.9238
- End learning rate: 1.0000 × 10^−9^

### 3.5. Data Preprocessing

Two methods have been used to preprocess the data

The first one is called feature scaling, these methods do not aim to extract new information from the data, it changes the scale of it only.

The user’s biofeedback signal is extracted on a server that is monitoring human health conditions based on emerging wireless mobile technologies with wireless body sensors. Different datasets have been used for the experiments. The first contains EMG signals with two target classes: normal and aggressive. This task is considered a time series data classification problem.

Table 2 shows the EMG physical action dataset includes 4 different patients, performing 10 different actions, each patient is classified as binary normal or abnormal.

The second method produces new features from the data such as RMS. We tested the two methods with different models and this section needs more investigation.

- Feature Scaling:
- Standard Scaling:

Standardize features by removing the mean and scaling to unit variance.

This is a 2D vis. For the effect standard scaling at 8 channels of Subject 1 at normal state while handshaking.

- Robust Scaling

Scale features using statistics that are robust to outliers.

- Min–Max Scaling

It essentially shrinks the range such that the range is now between 0 and 1 (or −1 to 1 if there are negative values).

- Normalizer

**Figure 11:**
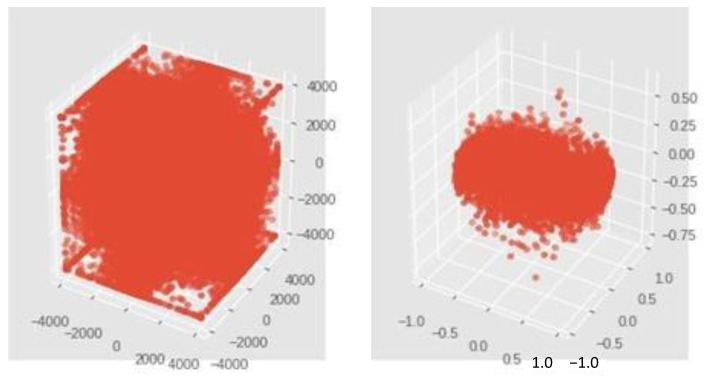
This is a 3D vis. For the effect normalization at 3 channels of Subject 1 at normal state while handshaking. The aim of this figure is to picture the way the data are pre-processed by representing three dimensions out of eight.

The normalizer scales each value by dividing each value by its magnitude in n-dimensional space for n number of features (Figure 11). This is a 3D vis. For the effect normalization at 3 channels of Subject 1 at normal state while handshaking.

Wavelet transforms are some of the more efficient techniques for processing nonstationary signals such as biomedical signals (e.g., EMG). Wavelet transforms the signal into its time–frequency domains. There are two types of wavelet analysis, discrete wavelet transform (DWT) and continuous wavelet transform (CWT). Figure 11. The subfigure on the left corresponds to feature scaling process while the subfigure on the right corresponds to standard scaling. Standardize features by removing the mean and scaling to unit variance in order to process the signals easier.

Both of them consume little time for signal processing. CWT is more consistent, but DWT has proven efficiency in analyzing nonstationary signals, although it yields a high-dimensional feature vector. In our research, discrete wavelet transform (DWT) is used for analyzing the EMG signal and extracting significant features which are very useful in identification of healthy, myopathic, and neuropathic subjects. 

**Figure 12:**
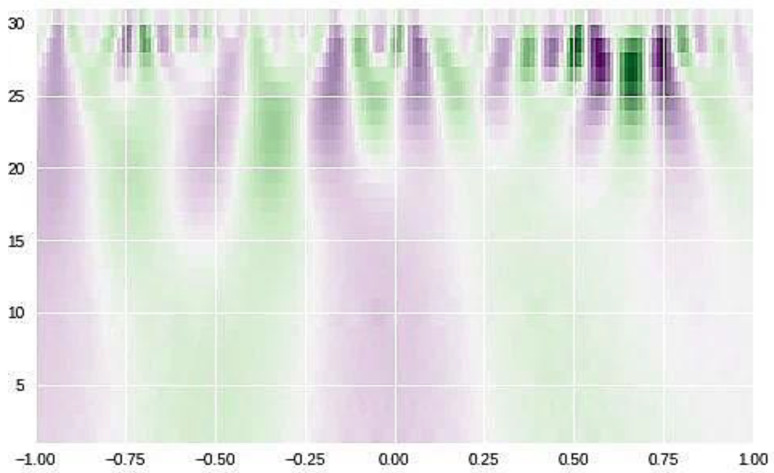
EMG MAV feature window size and values sample.

Seven features of the EMG signal are taken into consideration in this research. Root mean square (RMS), mean absolute value (MAV), zero crossing (ZC), slope sign change (SSC), and standard deviation (SD). Each one of these features is used as input to the classification process which is the next phase after the feature extraction process. The EMG MAV feature window size and values sample are presented in Figure 12.

**Figure 13:**
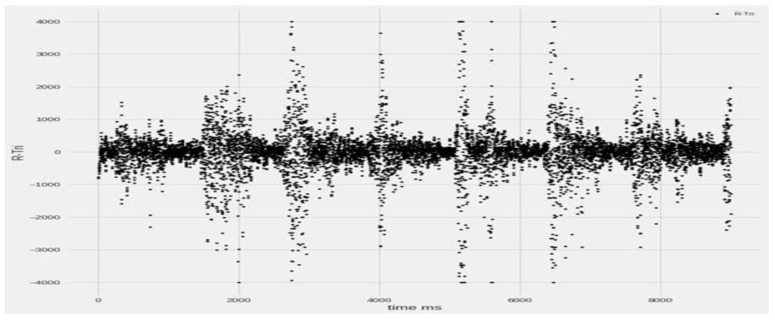
Real-time EMG muscle sample raw data in time ms [14,15,16].

A Daubechies wavelet function [34] of degree four (db4) was applied on each frame of the EMG signals in training and testing data so that the next step is to extract time and time–frequency features from the resulting processed signal (Figure 13).

This will be the main expert system engine for suggested initial diagnosis and emergency calls to the nearest hospitals for overall patient management and safety. It will depend on neural networks and case-based reasoning technologies.

**Figure 14:**
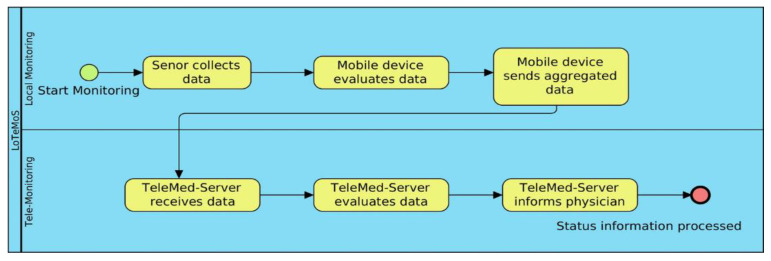
Mobile sensing, sensor collects data, mobile device, and telemedicine server.

The first step in our system is the sensor collects data. Mobile sensing process is shown in Figure 14, where mobile device evaluates data. Then, mobile device sends aggregated data to the telemedicine server. Then, telemedicine server evaluates data and informs physicians about our upcoming developments in artificial intelligence expert system. 

**Figure 15:**
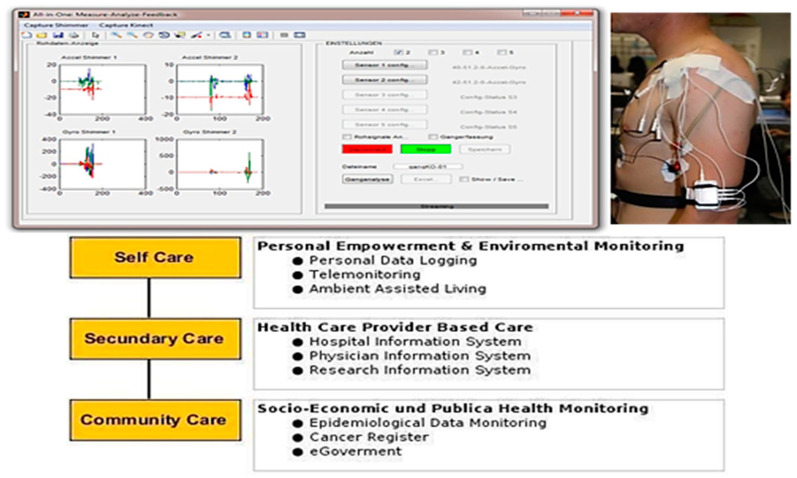
Real-time patient EMG data acquisition process by shimmer sensor [14,15,16].

In this research, analyzing data of stroke based on EMG sensors, as shown in Figure 15 of muscle readings to enable extracting best features. Then, significant features for efficient classification are selected since it determines the success of the pattern classification system. However, it is quite problematic to extract the best feature parameters from the EMG signals that can reflect the unique feature of the signal to the motion command perfectly. Hence, multiple feature sets are used as inputs to the EMG signal classification process. Some of the features are classified as time domain, frequency domain, time–frequency domain, and time-scale domain; these feature types are successfully employed for EMG signal classification. The next step is the signal classification phase.

The data acquisition process (Figure 15) consists of both real-time methodology as experiments conducted by German researchers at the Brandenburg University [14,15,16], and comparison to the offline dataset of the UCI and the Ain Shams University researchers [3] for different EMG signals channels samples, different colors, at different scaling.

The EMG signals are known for their uniqueness in every subject. An EMG sample consists of five channels:

(1) RF: Rectus Femoral
(2) BF: Biceps Femoral
(3) VM: Vastus Medial
(4) ST: Semitendinosus
(5) FX: Knee Flexion

Several feature combinations have been tested for obtaining the optimal signal results, which total 128 features. The number of classes of motions is 20, which consists of 10 normal and 10 aggressive physical actions. 

The following are normal: bowing, clapping, handshaking, hugging, jumping, running, seating, standing, walking, and waving. the following are aggressive: elbowing, front-kicking, hammering, heading, kneeing, pulling, punching, pushing, side-kicking, and slapping.

**Figure 16:**
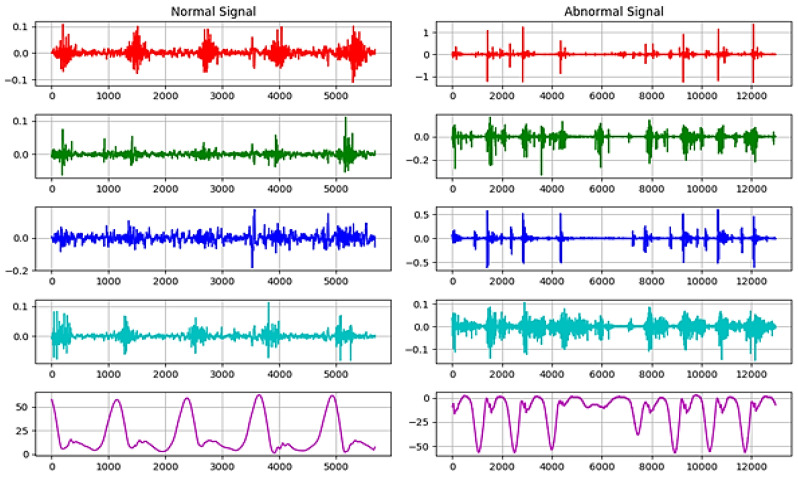
Real-time EMG signals sample histogram of normal and abnormal signals for different EMG signals channels samples at different scaling.

Figure 16 shows a sample of normal and abnormal EMG signals [14,15,16]. Seven features have been selected to obtain optimal results in signal classification. A sample of extracted features with the final selected features is shown in Table 3.

**Table 3:** Final selected features.

Abv.	Name of Feature	Definition
IEMG	Integrated EMG	$IEMG=\sum_{i=1}^{N}\left|X_{i}\right|$
MAV1	Modified mean absolute value type 1	$MAV1=\frac{1}{N}\sum_{i=1}^{N}w_{t}\left|X_{i}\right|,\;\;\;w_{t}=\{\begin{array}{c} 1^{}\;\mathrm{if}\;0.25N\leq i\leq 0.75N\\ 0.5,otherwise \end{array}$
RMS	Root Mean Square	$RMS=\sqrt{\frac{1}{N}\sum_{i=1}^{N}X_{i}^{2}}$
AAC	Average Amplitude Change	$AAC=\frac{1}{N}\sum_{i=1}^{N-1}\left|X_{i+1}-X_{i}\right|$
ZC	Zero Crossing	$ZC=\sum_{i=1}^{N-1}\left[\mathrm{sgn}\left(X_{i}\times X_{i+1}\right)\cap \left|X_{i}-X_{i+1}\right|\geq treshold\right]$
WAMP	Willison Amplitude	$WAMP=\sum_{i=1}^{N-1}\left[f\left(\left|X_{n}-X_{n+1}\right|\right)\right]$
WL	Waveform length	$WL=\sum_{i=1}^{N-1}\left|X_{i+1}-X_{i}\right|$

Integrated EMGIEMModified Mean Absolute Value 1MAV1Root Mean SquareRMSAverage amplitude changeAACZero crossingZCWillison amplitudeWAMPWaveform lengthWL

The following datasets were used:

Dataset Name: EMG lower limb dataset

The EMG lower limb dataset includes different 24 patients, performing three different actions, each patient is classified as binary normal, and abnormal.

Dataset Name: mHealth dataset

The mHealth dataset includes different 10 subjects performing different 12 actions, it also includes different measurements of subject kinematic information.

Both of the datasets are being used in the experiment for evaluating the model speed and accuracy in handling case-by-case. One patient sends two signal channels per time: the EMG signals reading and the ECG signals reading.

So, each patient has a multi-dimensional vector describing his input 𝑋 = [EMG Signal Samples, ECG Signal Samples].

## 4. Results

Accuracy reached 85% with the following characteristics: A learning rate of 𝐿 = 0.0001

Binary Cross Entropy Loss (BCE) function for loss measurement. The accuracy increased to 92% by using each supplied signal feature as a CNN input. The total test time was 5 s for 4 test subjects and there were a total of 24.576 test signal samples. 

There are two approaches for distributing deep learning models on the cloud, as shown below.

1. Model Parallelism:

In this paradigm, there is only one model distributed on different machines or different GPUs. For example, different layers may be assigned to different machines. This paradigm is useful for big models.

2. Data Parallelism:

In this paradigm, the model is copied between more than one machine. Each model runs on a different subset of the data.

**Figure 17:**
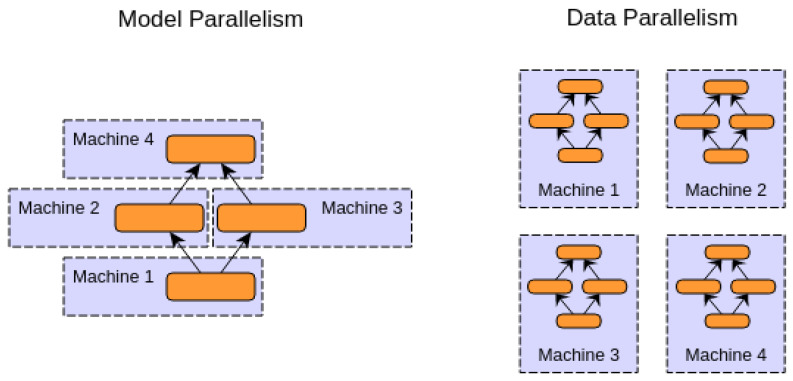
Model and data parallelism.

Figure 17 shows a graphical explanation of the two paradigms. All computations are completed based on model parallelism. 

The following properties of the GMDH-type polynomial network have been used during the test:
Activation FunctionLinearSelection CriterionValidate and BiasEpsilon Training Error0.001

The proposed mobile AI smart hospital platform consists of two main parts, the first one uses the stack CNN as AI cloud-based and the other GMDH and LSTM modules are used for the mobile AI app. For the first one, its main objective is presented as a new real-time CNN-stroke and Stroke and BAN-IOT: a deep learning model for signal deep feature extraction and classification within a cloud streaming environment. First, the use of stacked CNN is for handling the big data streaming of several signals sent from wearable sensors and body area networks (BAN) that include a variety of signals that do not correlate with each other on a shallow feature level. Scenario: Table 4 shows a sample of the EMG 8 channels.

- EMG Signal
- ECG Signal
- BAN Signal
- IMU Signal

Case Characteristics:

One patient sends two signal channels per time: 1—EMG signals reading

2—ECG signals reading

So, each patient has multi-dimensional vector describing his input

*X* = [EMG Signal Samples, ECG Signal Samples]

This model has been tested on mHealth [ref.] dataset, and on the EMG lower limb dataset.

Accuracy increased to reach 92% by using each supplied signal feature as a CNN input.

Total test time = 5 s for 4 test subjects

Total test signal samples = 24.576 signal samples

Concerning the processing time of GMDH it has taken nearly 30 min for the training. 

(2) The evaluation method has tested different modules for the overall new smart hospital platform, as AI-based software implementation only. 

Performance and results on cloud platform (GCP): Table 5 shows the performance and validation accuracy that has been achieved in our experiments, when implementing the stacked CNN, using two Nvidia GPUs. The use of AI-based cloud simulating the smart hospital platform. 

Tests have been conducted on 10 different physical action signals, the test samples have been divided almost evenly, 5k samples for training and ~5k samples for testing the prediction.

Table 6, below, shows the characteristics and scores for a selected subject, where 10 aggressive different actions are predicted using the GMDH-type neural network.

On average, among the selected subjects the model is able to predict accurately 96.02% of the signal in low time.

The table below shows the characteristics and scores for the previously selected subject, 10 normal different actions are predicted using the GMDH-type neural network.

As shown in Table 6, different experiments have been conducted by training the GMDH deep learning model on different input sizes of EMG channels. As illustrated different layers have different accuracies, according to input sizes of EMG 4 channels, EMG 8 channels, and EMG of mHealth dataset. On average, the selected subject’s model is able to predict accurately 96.85% of the signal in low time.

The following plots demonstrate two selected actions from the previously conducted test experiments, 1 normal action, and 1 aggressive action. 

As shown in Table 7, different experiments have been conducted by training the GMDH deep learning model on different input sizes of EMG channels. As illustrated different layers have different accuracies, according to input sizes of EMG 4 channels, EMG 8 channels, and EMG of mHealth dataset. 

The graphs from Figure 16 show the following: original signal in red, read from the EMG sensor; predicted signal in orange, using the GMDH-type neural network; overlapped signals, to show prediction visual accuracy, in both blue and green, and finally, the cross-correlation between the two signals. It can be clearly seen how visually both signals (predicted and original) are almost equal.

**Figure 18:**
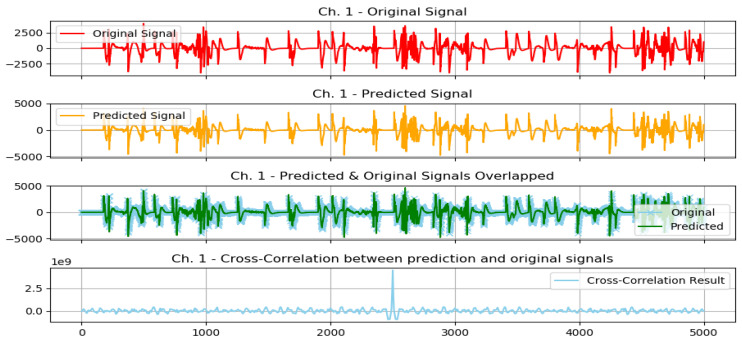
Plot of aggressive action for selected subject.

The graphs from Figure 18 show the following: original signal in red, read from the EMG sensor; predicted signal in orange, using the GMDH-type neural network; overlapped signals, to show prediction visual accuracy, in both blue and green, and finally, the cross-correlation between the two signals. It can be clearly seen how visually both signals (predicted and original) are almost equal.

**Figure 19:**
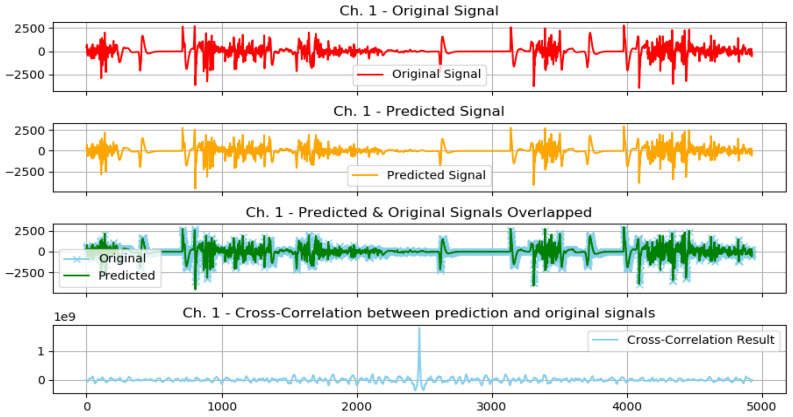
Plot of normal action for selected subject.

We show the same graph but for a normal action below (Figure 19). 

In addition, based on the previous plot it can be clearly seen how visually both signals (predicted and original) are almost equal. 

**Figure 20:**
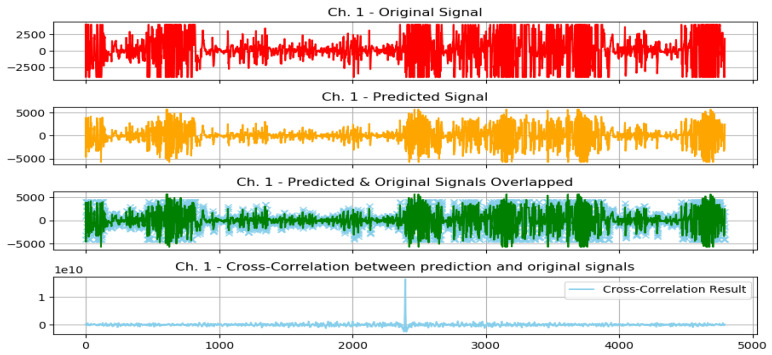
Signal prediction closer results: sample aggressive action prediction.

**Figure 21:**
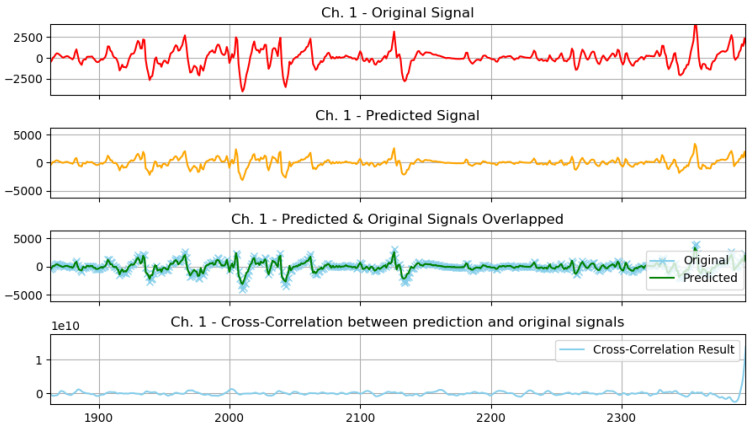
A closer look at the signal prediction details.

The previous graph shows a prediction sample of an aggressive action (Figure 20), and the following graph shows a closer look at the signal prediction details (Figure 21).

It can be clearly seen how the signal-predicted values are close to the original values, most of the values differ by a small value that will not change the behavior of the signal when analyzed, thus preserving the information needed for further analysis and classifications.

Next, we will show each subject test scores and then show a generalized average score for the model with training statistics.

The aggressive action/normal action test scores for Subject 1/Subject 2 are shown in Table 8, Table 9, Table 10 and Table 11.

**Figure 22:**
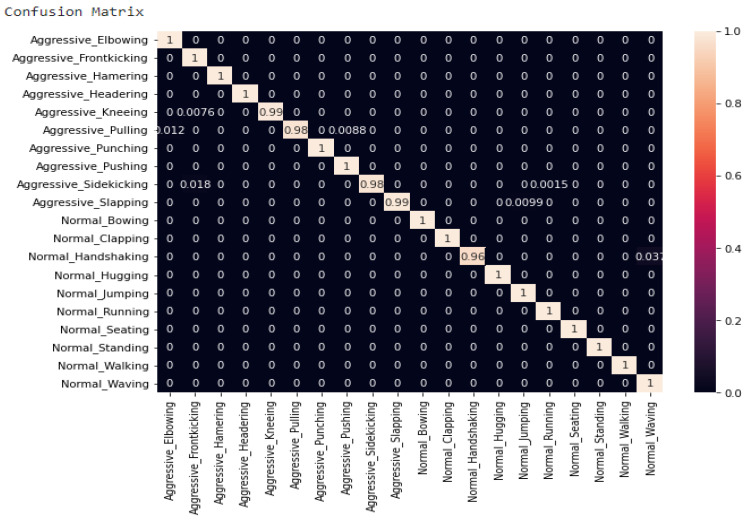
The confusion matrix generated.

**Figure 23:**
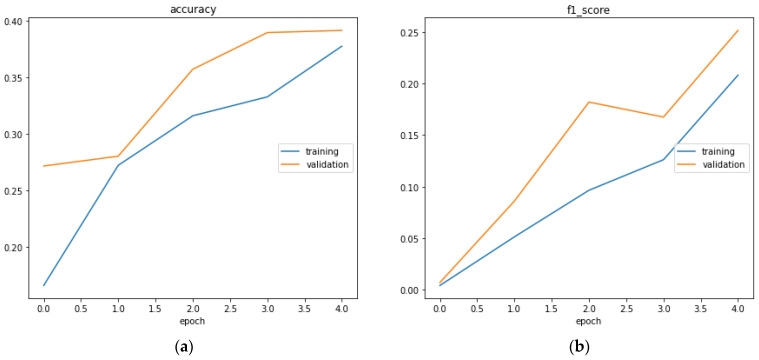
The accuracy evaluation (**a**) and f1 score over epochs (**b**).

The confusion matrix of the GMDH model is shown in Figure 22 is generated. Different metrics generated by the neural network of results are shown in Figure 23. This is to show for classification of whether the action is aggressive or normal a combination of results between normal action test scores and aggressive action test scores in order to more clearly see the results from these two cases. Additionally, the classification report represented below helps us to better understand the metrics taken into consideration for this example (Table 12).

In Figure 23, section (a) presents the accuracy evaluation and (b) presents f1 score

$$F=2\cdot \frac{precision\cdot recall}{precision+recall}$$

$$Accuracy=\frac{tp+tn}{tp+tn+fp+fn}$$

**Figure 24:**
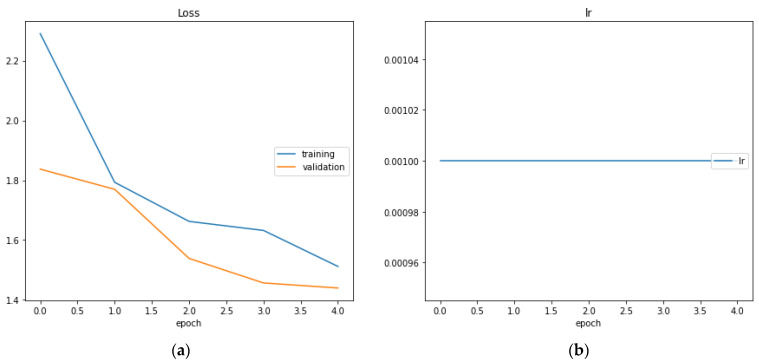
The loss function (**a**) and the learning rate (**b**).

In Figure 24, section (a) the loss function is represented while in section (b) the learning rate is represented. We can see that the learning rate is constant and equal to 10^−3^.

**Figure 25:**
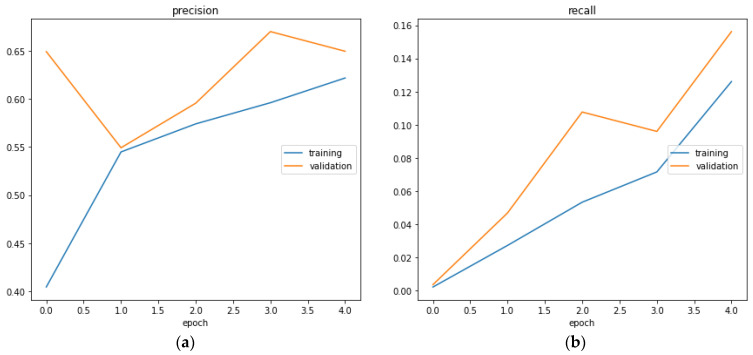
The precision (**a**) and the recall (**b**) represented by epochs.

In Figure 25, the precision is represented regarding epochs (a) and in section (b) the recall is presented also regarding epochs.

$$Precision=\frac{tp}{tp+fp}$$

$Recall=\frac{tp}{tp+fn}$, where *tp* = true positive, *tn* = true negative, *fp* = false positive, *fn* = false negative

**Figure 26:**
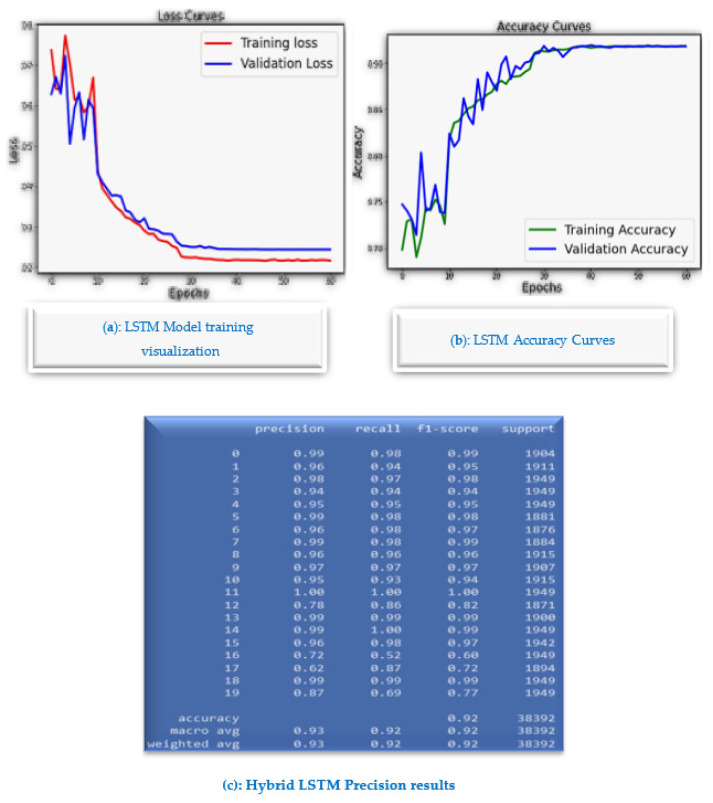
The parallel LSTM method (**a**) visualization of LSTM model training, (**b**) LSTM accuracy curves, (**c**) Overall results of LSMT hybrid model.

In contrast to many GMDH deep learning-based approaches, the proposed algorithm is lightweight for the proposed Mobile AI Engine, and therefore, brings continuous diagnosis and prediction with accurate GMDH-LSTM-based EMG signal prediction to IoMT simulated inputs. The highest precision of parallel LSTM achieves 99.9% and the average reaches 93.65%. Figure 26a shows LSTM Model training visualization, while Figure 26b shows LSTM accuracy curves and Figure 26c shows the overall hybrid LSMT model results. The main advantage of the parallel LSTM model is that it is more stable than the GMDH deep learning model, as it has been trained on the 8 EMG channels directly. In addition, the parallel LSTM model is more suitable for the implementation of the mobile health app. However, the GMDH deep learning is good as a standalone predictive model for mathematical modeling EMG signal predictions. In contrast to many GMDH deep learning-based approaches, the proposed algorithm is lightweight for the proposed mobile AI engine, and therefore, brings continuous diagnosis and prediction with accurate GMDH–LSTM-based EMG signal prediction to IoMT simulated inputs. The highest precision of parallel LSTM achieves 99.9% and the average reaches 93.65%. The main advantage of the parallel LSTM model is that it is more stable than the GMDH deep learning model, as it has been trained on the 8 EMG channels directly. Additionally, the parallel LSTM model is more suitable for the implementation of the mobile health app. However, the GMDH deep learning is good as a standalone predictive model for mathematical modeling EMG signal predictions. The GMDH could predict most of the signals tested accurately with a high *R*^2^ score, below is a table of the general prediction scores tested on both normal and aggressive action signals.

It can be seen that the ability of the GMDH to predict more event-based signals with more peaks and more aggressive spectrum, is higher than normal signals with lower peaks and less aggressive spectrum.

The main disadvantages of the GMDH model are that: the peaks of the signal for the GMDH predictive model vary from channel to channel of the EMG signal. In addition, the GMDH training algorithm takes a lot of memory for training and may crash, it crashes at using memory above 13 GB and it is not suitable for real industry applications of the mobile AI health app. GMDH is a self-organizing approach by which gradually complicated models are generated based on the evaluation of their performances on a set of multi-input–single-output data. However, it is good for modeling the predictive analytics modeling of the stroke prediction system. It can be tested in the future for cloud computing or AI high-performance computing side. However, the hybrid parallel LSTM model is suitable for the mobile AI app implementation in python.

## 5. Discussion

The GMDH could predict most of the signals tested accurately with a high $R^{2}$ score, and below is a table of the general prediction scores tested on both normal and aggressive action signals (Table 13).

**Figure 27:**
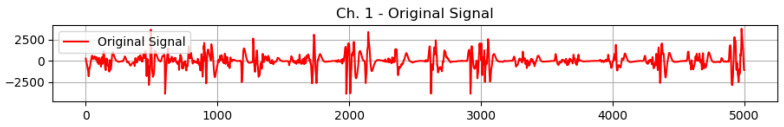
Normal action signal sample.

**Figure 28:**
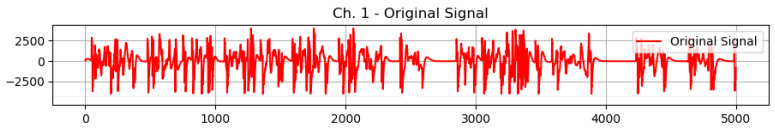
Aggressive action signal sample.

It can be seen that the ability of the GMDH to predict more event-based signals with more peaks and more aggressive spectrum is higher than normal signals with lower peaks and less-aggressive spectrum. Below are two different plots for a normal signal and an aggressive signal (Figure 27 and Figure 28).

Concerning the mobile GMDH algorithms analysis and time series data forecasting:

As we know time series data are a sequence of data taken in multiple time stamps. There are two main goals of processing time series data. The first task is to try to classify the data into predefined subcategories. The second task is to predict the future of the input data using the current data. There are multiple algorithms to process and forecast time series data (parametric and nonparametric algorithms). These include:

- Univariate time series forecasting
- Multivariate time series forecasting multi-step time series forecasting
- Deep networks based on the group method of data handling

GMDH networks are the first feedforward deep learning neural network (since 1969). It consists of a family of inductive algorithms that use automatically generated architecture and parametric optimization methods.

Given a training set, layers are incrementally growing by regression analysis, then pruned with help of a validation set. The number of layers and units can be learned in a problem-dependent fashion. When the architecture consists of a multilayer procedure, it becomes equivalent to the artificial neural network with a polynomial activation function of neurons.

GMDH only: We tried to follow the same conditions described in the previous report for training and testing but we faced some problems due to missing information in the report so we tried our best to reproduce the previous results which takes a lot of time and effort. We depended on the python implementation “GmdhPy”.

**Figure 29:**
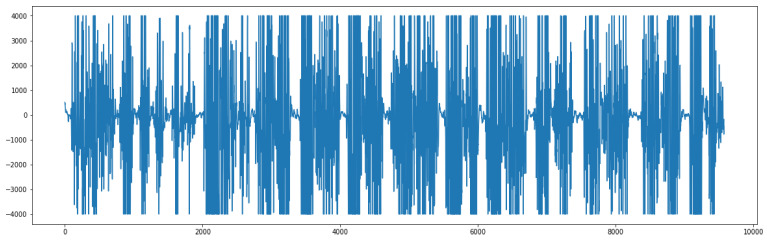
GMDH mobile AI signal sample prediction.

There are multiple ways we can define our inputs and our outputs given that we have four people. Every person has 20 actions given by 8 channels. At the same time, we have extracted 39 features from this data as described in previous reports. This gives us a huge space for experiments. We did not test all the possibilities, we only focused on the most obvious ones. We framed the problem as univariate time series forecasting. So the input is one channel for one subject. For example, we took the fourth subject with his first channel (RBic) and tried to predict the future of this channel as shown in Figure 29. 

There are multiple hyper-parameters that should be studied in the future such as how many readings from the past should we depend on and the hyperparameters of the GMDH.

We took 10 readings from the past and tries to predict the next one in the future.

As our scope was to develop a fast and stable model for the deployment we considered these settings:
*ref_functions = (‘linear_cov’),**criterion_type = ‘validate’,**criterion_minimum_width = 5,**stop_train_epsilon_condition = 0.001,**layer_err_criterion = ‘top’,**l2 = 0.5,**manual_best_neurons_selection = True,**min_best_neurons_count = 30,*

Calculating the root mean square error in time series data forecasting is very critical. So, we considered the time shift that happened in the data generation. So, GMDH achieved a train score of 1103.42 RMSE and a test score of 968.80 RMS

**Figure 30:**
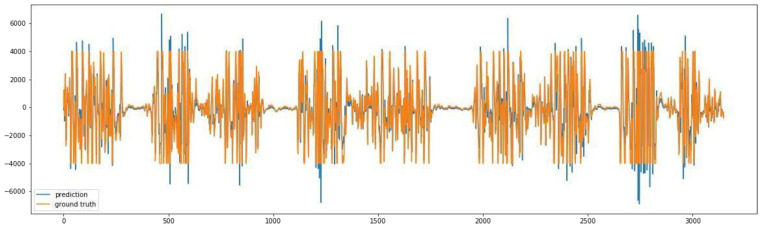
GMDH with predicted signal of higher peaks than the original signal when no residual connections or previous time steps are included.

**Figure 31:**
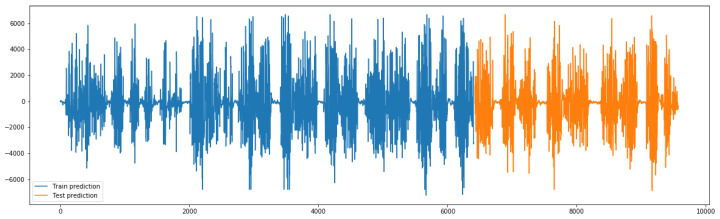
GMDH with predicted signal of better results of predicted signal with residual connections or previous time steps are included.

We see that the predicted signal has higher peaks than the original signal as shown in Figure 30. The problem with the GMDH-only setup is that the network did not keep the previous information from the previous time steps or residual connections. As described in this paper, when GMDH is combined with some information from the previous time steps it can give better performance with residual connections results as shown in Figure 31.

Training and testing:

The number of subjects samples is four subjects, three male and one female. Three of them are because Subject 2 has noisy data. 

**Figure 32:**
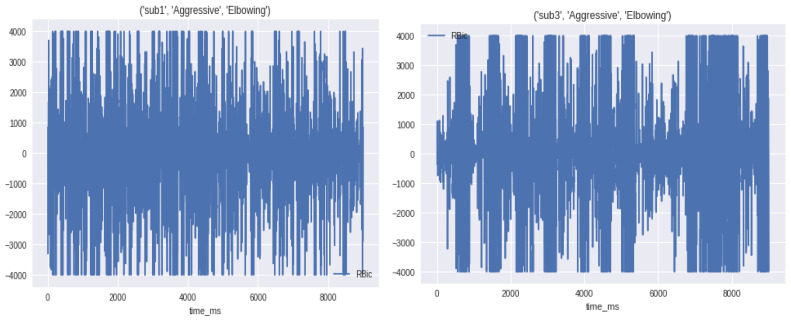
EMG signals aggressive class gives high peaks during analysis.

We found that there is a pattern between the action and the aggressiveness, for example, when you are running this state is classified as normal. The upper body returns normal signals but the lower body returns high peak signals. Although in the aggressive class, for example, the lower and upper body return relatively high peak signals, as illustrated in Figure 32.

**Figure 33:**
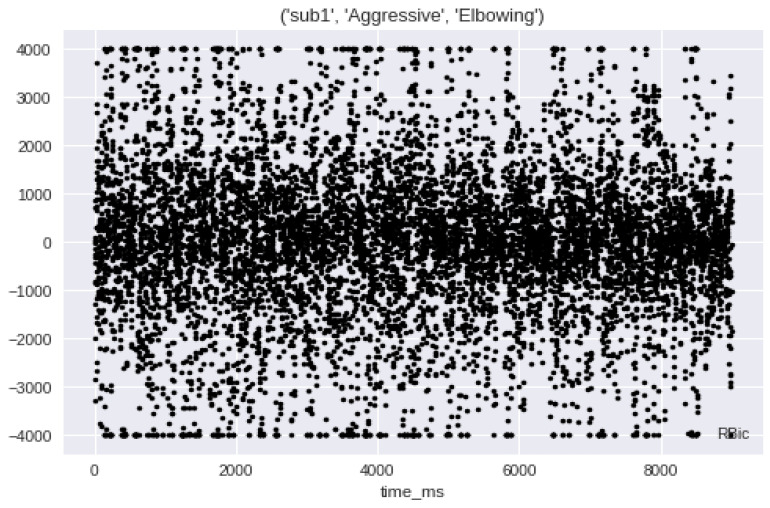
EMG signals of all 8 channels have the same pattern of highly distributed points during elbowing motion examples.

In Figure 33, the subject is in an aggressive move, he is doing an elbowing move. We found that all eight channels have the same pattern of highly distributed points in all the graphs with some concentration in the middle.

Long Short-Term Memory Network (LSTM):

Maintain state (memory) across very long sequences. Temporal information was used because LSTM is very sensitive to the data ranges we applied. Data normalization and scaling were used in the input and output. We used standard scaling for the input. It can be solved using linear activation in the output layer.

A novel stroke prediction algorithm is proposed based on EMG signals prediction. The proposed solution employs 612 a novel architecture consisting of a group method of data handling and multiple LSTM recurrent neural networks. Results: Experimental evaluations show superior EMG prediction performance compared to previous works. Measurements with different deep learning methods as combining CNN with LSTM show that the proposed algorithm meets performance requirements for continuous and real-time execution on IoMT devices. Conclusion: In 616 contrast to many compute-intensive deep learning-based approaches, the proposed algorithm is lightweight for the proposed mobile AI engine, and therefore, brings continuous diagnosis and prediction with accurate GMDH–LSTM-based EMG signal prediction to IoMT simulated inputs.

Mobile open architecture. We adopted an open architecture to make it easy for any researcher. They will be able to add their own model to the mobile in an easy way. Most mobile apps are currently closed systems, meaning that the researcher cannot add his/her model without many changes. Any model can be added instead of our trained models.

Findings: The first idea about the data, it has a huge variance and the data are not zero, meaning there are two levels of classification. The first level is normal or aggressive. This level depends on the activities which the subjects are doing to differentiate between the two classes. In the second level, for every state from level 1 (normal or aggressive), the subject was doing 10 activities for normal and 10 activities for aggressive.

To conclude, the signal for both classes normal and aggressive was processed and there were observed differences that helped us extract characteristics such as integrated EMG, modified mean absolute value 1, root mean square, average amplitude change, zero crossing, Willison amplitude, and waveform length. Great results were obtained by using a deep learning model, the new GMDP deep learning model. 

Bearing all these things in mind, a model that can detect these two sides of behavior may help people that suffered a stroke to communicate easier and interact more with the environment. Even though this study was intended to help patients of a hospital, there may be applications in the security domain field in order to predict aggressive behavior.

In addition, a new hybrid LSTM/dense deep learning architecture has been added with detailed experimental results for EMG stroke prediction and as compared to GMDH, it is better as a parallel model that takes as input all the EMG 8 channels with high results; however, the GMDH algorithm can be easily deployed as mobile AI app with high accuracies. More results need to be tested in future work for parallel inputs to the GMDH algorithm. However, both models achieve high stroke prediction accuracies. Additionally, a single LSTM module has been integrated into the GMDH algorithm for enhancing the mobile AI implementation for stroke prediction.

## 6. Conclusions and Future Work

Artificial intelligence (AI) technologies in smart health patients’ safety and care for connected health and intelligent diagnostics and predictive health ai edge computing integrated within smart hospital environments, have opened up new opportunities in healthcare systems and complex disease predictions and early detection for issues such as heart and stroke diseases. Smart hospital technologies [21,22,23,24,25,26,27] are a steadily growing field in artificial intelligence (AI), biomedical big data analytics [44,45,46,47] Internet of Medical Things (IoMT). Heart diagnosis and stroke prediction have urgent patient cases that may cause problems such as cardiovascular diseases [28,29,30,31], heart attacks, and brain strokes. It may also cause sudden death. These are the leading cause of death in the Middle East, Europe, and the United States. For these reasons, heart and stroke diseases are considered emergency cases. In the recent research of artificial intelligence technologies in the healthcare domain, what we witness is high competition and new revolution [1,2,3]. However, today’s AI research and development of technologies in the fields of heart diseases diagnosis [16,17,18,19,20] and stroke prediction research are still missing a real-time AI-based heart diagnosis and stroke prediction system to be developed as AI-based platform R&D to be used in the industry and the new era of smart hospital developments [21,22,23,24,25,26,27]. This research paper innovation introduces a new AI system design that consists of an integrated real-time IOT-AI smart heart/stroke platform to be in the future inside hospitals as a new IoMT-AI-based heart/stroke platform and as an independent mobile AI telemedicine system for stroke prediction. Artificial intelligent IOT hospital edge-connected health diagnostic and predictive systems integrated with telemedicine services for both elder patients with chronic and brain stroke cases aim to help heart/brain stroke patients to discover their disease once it occurs based on EEG/ECG/EMG signals. However, classifying real-time ECG/EMG signals [14,15] is a complex task, especially for patient muscle signal feedback problems. This proposal introduces an integrated artificial intelligence telemedicine platform including AI software for heart disease diagnosis, and AI software for brain stroke diagnosis and prediction. The highest precision of parallel LSTM achieves 99.9% and the average reaches 93.65%. AI/DL telemedicine services could be useful for the nearest hospital and patients’ telemonitoring at-home care services. In this research paper, we have only presented some innovative research results for the full mobile AI system cycle, and some real implementations in simulated tests. Our solution is more innovative than previous research on stroke prediction using only single deep learning or some sample stroke cases such as during sleeping, as discussed previously in the paper in Section 2.

This research article also presents an overall state-of-the-art artificial intelligence mobile health system architecture for stroke that can be implemented by AI and IoT companies such as Dell technologies for real-life scientific implementations. The main focus is on predictive analytics and edge computing solutions in healthcare and emergency situations.

Predictive analytics deals with information retrieval to predict an unknown event of interest, typically a future event. Using technology that learns from data to predict these unknown events could drive better decisions. This research paper utilizes the concepts of deep learning (GMDH) for signal predictions for mobile edge computing future implementations of complete solutions in a smart health home living scenario.

This research paper has successfully presented several steps in the predictive analytics process: identification of the problem and a determination of the outcomes and objectives is a crucial first step. The first data of the model used EMG from both real-time and offline datasets.

Future work may include different ways to process the signal, and data processing time concerning the processing time of GMDH it has taken nearly 30 min for the training, but the networking connections are out of the scope of this article and we are honored to present them in the future work of next phase of app implementation. Additionally, other deep learning model architectures will be presented in order to achieve better precision in classification. In addition, for the XAI decision-based tools, extended parts of TIER 2 and TIER 3 of fuzzy logic and surveillance-based systems will be considered for future works. Additionally, future work may include different ways to process the signal, and other deep learning model architectures in order to achieve better precision in classification.

## Figures and Tables

**Table 1:** Network time measurements.

Operation	Time Cost (Seconds)
Feedforward during training	0.152 s
Total Training for 500 Epochs	240 s (4 min)
Sample Extraction and Prediction	0.025 s
10 million Samples on 8 Nodes	25 s per Node

**Table 2:** EMG physical action dataset specifications.

Signal Type	Time Series
Number of Instances per Channel	~10,000 Sample
Number of Channels	8 Channels
Number of patients	4 patients

**Table 4:** Shows a sample of the EMG 8 channels data included in the dataset [48].

Channel 1	Channel 2	Channel 3	Channel 4	Channel 5	Channel 6	Channel 7	Channel 8
−8.3532	2.5062	−2.406	−1.821	−1.1889	0.39931	−9.4774	−2.199
−7.073	2.1373	−2.6182	−2.0345	−1.3689	1.0842	−8.8761	−1.4746
−7.6434	2.3723	−2.4497	−2.2773	−1.4443	2.2408	−9.8723	−1.0962
−8.0995	2.4517	−2.7688	−2.4155	−1.4903	1.8016	−9.8628	−1.1314
−8.3307	2.3913	−3.0164	−2.1852	−1.394	0.44444	−9.9628	−1.2333
−8.7935	2.8082	−2.7473	−1.9383	−1.3229	1.0435	−9.7058	−1.2769
−8.9454	2.8268	−2.7351	−1.6787	−1.1889	1.8488	−9.6066	−1.2535
−9.4027	2.9628	−2.6491	−1.4568	−1.0424	1.6883	−9.5739	−0.95139
−8.9815	3.116	−2.4038	−1.4401	−1.0382	1.2073	−10.205	−0.69609
−9.1775	2.8138	−2.6836	−1.4903	−1.0173	1.1904	−10.231	−0.37467
−8.64	2.7076	−2.63	−1.4819	−0.90424	1.2918	−9.992	−0.19502
−8.7625	3.7808	−3.0071	−1.0717	−0.55259	1.8689	−9.5166	−0.17589
−8.5805	3.7717	−2.9237	−0.7619	−0.40607	1.9708	−9.4467	−0.1193
−8.6582	3.6193	−2.9164	−0.51073	−0.20513	1.7172	−9.9153	−0.43692
−8.6379	3.6099	−2.9593	−0.29304	−0.029304	1.2985	−9.9256	−0.4833
−8.4253	3.5899	−2.7716	−0.15489	0.066981	1.0929	−9.9872	−0.69676

**Table 5:** The performance and validation accuracy for the stacked CNN-cloud-based experiments.

Framework	Time to Train	Inference Time	Achieved Validation Accuracy	Number of GPUs	Scaling Efficiency
Keras	2 min	40 μs	98%	1	-
Keras + Tensoflow	2.3 min	45 μs	97.96%	2	88.88%

**Table 6:** Model average prediction accuracy on the selected subjects was 96.85%.

Channels	Layers	RMSE	NRMSE	R-Squared	Train Time (s)	Test Time (s)
EMG 4 channels	7	355.257	0.039	98.60%	0.08924	0.000844717
EMG 8 channels	7	96.517	0.013	97.61%	0.08548	0.000657558
EMG m health channels	7	134.699	0.015	97.47%	0.07976	0.000873566
EMG 4 channels	3	29.893	0.007	97.23%	0.04030	0.000925303
	2	14.610	0.009	96.78%	0.01175	0.000540018
EMG 8 channels	3	61.059	0.011	96.41%	0.02851	0.000662088
	2	2.203	0.032	96.15%	0.01576	0.000739336
EMG m health channels	3	233.189	0.025	96.13%	0.03011	0.000577211
	2	27.089	0.026	96.08%	0.01697	0.000613451
	50	148.183	0.014	95.99%	0.59829	0.000548363
	Averages	110.27012	0.0191853	96.85%	0.09962	0.000698161

**Table 7:** On average, among the selected subjects the model is able to predict accurately 96.02% of the signal in low time.

Channels	Layers	RMSE	NRMSE	R-Squared	Train Time (s)	Test Time (s)
EMG 4 channels	7	232.583	0.028	97.12%	0.07720	0.00047
	2	171.787	0.018	96.98%	0.01279	0.00055
EMG 8 channels	7	220.009	0.024	96.96%	0.08461	0.00090
EMG m health channels	7	204.746	0.025	96.85%	0.10477	0.00059
	3	191.501	0.020	96.68%	0.02523	0.00074
	2	213.642	0.021	96.06%	0.01341	0.00082
EMG 4 channels	3	265.888	0.027	95.94%	0.02851	0.00083
	7	304.756	0.031	95.43%	0.09501	0.00066
EMG 8 channels	3	262.885	0.027	95.34%	0.03835	0.00100
EMG m health channels	3	347.536	0.032	92.82%	0.03151	0.00095
	Averages	241.53331	0.0252804	96.02%	0.05113833	0.000750542

**Table 8:** Aggressive action test scores for Subject 1, the overall average reaches 90.01%, after training of adjusting different layers with different EMG signals channels as used before in Table 4 and Table 5, respectively.

Subject 1					
Layers	RMSE	NRMSE	R-Squared	Train Time (s)	Test Time (s)
3	522.285	0.043	86.46%	0.03084	0.00056
2	148.729	0.024	90.79%	0.01768	0.00083
2	552.910	0.036	90.79%	0.01298	0.00066
2	48.896	0.027	91.09%	0.01208	0.00059
7	234.463	0.027	90.15%	0.08429	0.00067
2	621.693	0.040	92.03%	0.01433	0.00072
7	491.597	0.038	89.49%	0.09936	0.00037
3	424.720	0.036	89.46%	0.04247	0.00083
3	209.147	0.023	87.80%	0.02835	0.00093
2	370.014	0.030	92.07%	0.02202	0.00114
Averages	362.4453	0.0324189	90.01%	0.03644128	0.000729

**Table 9:** Normal action test scores for Subject 1, the overall average reaches 81.43%, after training of adjusting different layers with different EMG signals channels as used before in Table 4 and Table 5, respectively.

Subject 1					
Layers	RMSE	NRMSE	R-Squared	Train Time (s)	Test Time (s)
3	7.683	0.019	95.33%	0.03059	0.00088
7	110.723	0.024	90.46%	0.09436	0.00065
3	205.429	0.022	89.36%	0.02513	0.00083
2	84.455	0.029	89.12%	0.01444	0.00089
7	85.226	0.020	87.21%	0.10529	0.00076
3	193.760	0.036	86.41%	0.03894	0.00092
2	99.943	0.051	74.39%	0.01358	0.00158
2	22.388	0.112	68.79%	0.01468	0.00144
3	7.941	0.196	66.66%	0.02479	0.00083
2	7.983	0.171	66.57%	0.02555	0.00075
Averages	82.55306	0.067997	81.43%	0.03873329	0.0009532

**Table 10:** Aggressive action test scores for Subject 2, the overall average reaches 96.2%, after training of adjusting different layers with different EMG signals channels as used before in Table 4 and Table 5, respectively.

Subject 2					
Layers	RMSE	NRMSE	R-Squared	Train Time (s)	Test Time (s)
3	262.885	0.027	95.34%	0.03835	0.00100
7	232.583	0.028	97.12%	0.07720	0.00047
7	304.756	0.031	95.43%	0.09501	0.00066
2	171.787	0.018	96.98%	0.01279	0.00055
7	220.009	0.024	96.96%	0.08461	0.00090
3	191.501	0.020	96.68%	0.02523	0.00074
3	347.536	0.032	92.82%	0.03151	0.00095
7	204.746	0.025	96.85%	0.10477	0.00059
3	265.888	0.027	95.94%	0.02851	0.00083
2	213.642	0.021	96.06%	0.01341	0.00082
Averages	241.53	0.0252804	96.02%	0.05113833	0.00075054

**Table 11:** Normal action test scores for Subject 2, the overall average reaches 96.68%, after training of adjusting different layers with different EMG signals channels as used before in Table 4 and Table 5, respectively.

Subject 2					
Layers	RMSE	NRMSE	R-Squared	Train Time (s)	Test Time (s)
7	355.257	0.039	98.60%	0.08924	0.00084
7	96.517	0.013	97.61%	0.08548	0.00066
7	134.699	0.015	97.47%	0.07976	0.00087
3	29.893	0.007	97.23%	0.04030	0.00093
3	61.059	0.011	96.41%	0.02851	0.00066
2	2.203	0.032	96.15%	0.01576	0.00074
3	233.189	0.025	96.13%	0.03011	0.00058
2	27.089	0.026	96.08%	0.01697	0.00061
50	148.183	0.014	95.99%	0.59829	0.00055
7	252.557	0.028	95.10%	0.08081	0.00075
Averages	134.06	0.0210994	96.68%	0.10652237	0.00071871

**Table 12:** The classification report.

Action	Precision	Recall	F1-Score	Support
Aggressive_Elbowing	0.99	1.00	0.99	1881
Aggressive_Frontkicking	0.97	1.00	0.99	1861
Aggressive_Hamering	1.00	1.00	1.00	1949
Aggressive_Headering	1.00	1.00	1.00	1949
Aggressive_Kneeing	1.00	0.99	1.00	1964
Aggressive_Pulling	1.00	0.98	0.99	1921
Aggressive_Punching	0.99	1.00	1.00	1876
Aggressive_Pushing	1.00	1.00	1.00	1867
Aggressive_Sidekicking	1.00	0.98	0.99	1953
Aggressive_Slapping	1.00	0.99	1.00	1926
Normal_Bowing	1.00	1.00	1.00	1915
Normal_Clapping	1.00	1.00	1.00	1949
Normal_Handshaking	1.00	0.96	0.98	1943
Normal_Hugging	1.00	1.00	1.00	1900
Normal_Jumping	0.99	1.00	1.00	1930
Normal_Running	1.00	1.00	1.00	1939
Normal_Seating	1.00	1.00	1.00	1949
Normal_Standing	1.00	1.00	1.00	1894
Normal_Walking	1.00	1.00	1.00	1949
Normal_Waving	0.96	1.00	0.98	1877
accuracy			1.00	38,392
macro avg	1.00	1.00	1.00	38,392
weighted	1.00	1.00	1.00	38,392

**Table 13:** Prediction scores.

Score/Action Set	Normal Actions	Aggressive Actions
RMSE	141.3881606	391.8395819
NRMSE	0.039652622	0.033451407
R-Squared	88.02%	90.54%

## Data Availability

Not applicable.
